# Descriptive analysis of the Application of Electroconvulsive Therapy in patients with acute psychiatric pathology admitted to a Psychiatric Hospitalization Unit

**DOI:** 10.1192/j.eurpsy.2025.2120

**Published:** 2025-08-26

**Authors:** M. F. Parada, R. Arias, I. Lizarraga, E. Chavarría, J. Jimenez, P. Fructos, C. Arranz, A. Ortiz, J. Bullard, M. Vallejo

**Affiliations:** 1 Psychiatry, Clinica Universidad de Navarra, Pamplona, Spain

## Abstract

**Introduction:**

**Electroconvulsive therapy (ECT)** is a recognized treatment for various psychiatric conditions (Bernardo et al., 2018). However, there is a lack of recent studies describing the clinical characteristics of patients undergoing ECT (Peltzman et al., 2020).

**Objectives:**

To provide an updated evaluation of the clinical **characteristics, treatment parameters, and outcomes** of patients receiving ECT at Clinica Universidad de Navarra (CUN), focusing on its **effectiveness and evolution** over recent years.

**Methods:**

This **cross-sectional descriptive study** examines patients who underwent electroconvulsive therapy (ECT) at CUN’s Psychiatric Hospitalization Unit from **January 2019 to August 2023.** It focuses on those who received bifrontotemporal ECT, with stimulation power quantified using the DGx program on the Thymatron® machine. Collected data include age, sex, prior psychotropic medication use, ECT indication, comorbidities, Stimulus potency (%DGx), seizure duration (seconds on the electroencephalogram), and anesthetic induction type. Hamilton Anxiety and Depression Scales were recorded before and after treatment when clinically indicated.

**Results:**

ECT was administered in 80 cases, constituting 8.62% of admitted patients, with 33.75% being male. Among these, 33% had **psychiatric comorbidities**, most commonly being pathological personality traits (16%) and generalized anxiety disorder (5.3%) (Image 1). **Non-psychiatric comorbidities** included endocrine-metabolic conditions 49% and cardiac conditions 34% (Image 2.)

Pre-ECT, the most **common psychotropic medications** included benzodiazepines (87.5%), atypical antipsychotics (76.5%), and dual antidepressants (47.5%). Propofol was used for **initial anesthetic induction** in 86.25% of cases, with 30.43% requiring a switch to thiopental due to the reduced efficacy. The average number of **ECT sessions** per patient was 8.9 (range: 3-13), with a **mean seizure duration** of 30.5 seconds. The **primary indications for ECT** were depressive disorders (85%) and psychotic disorders (11%) (Image 3).

Before ECT, the **average Hamilton Depression Scale** score was 25.3, decreasing to 5.3 post-treatment. Similarly, the **Hamilton Anxiety Scale score average** was 23.72 before ECT and 4.6 after.

**Image 1:**

**Figure:**
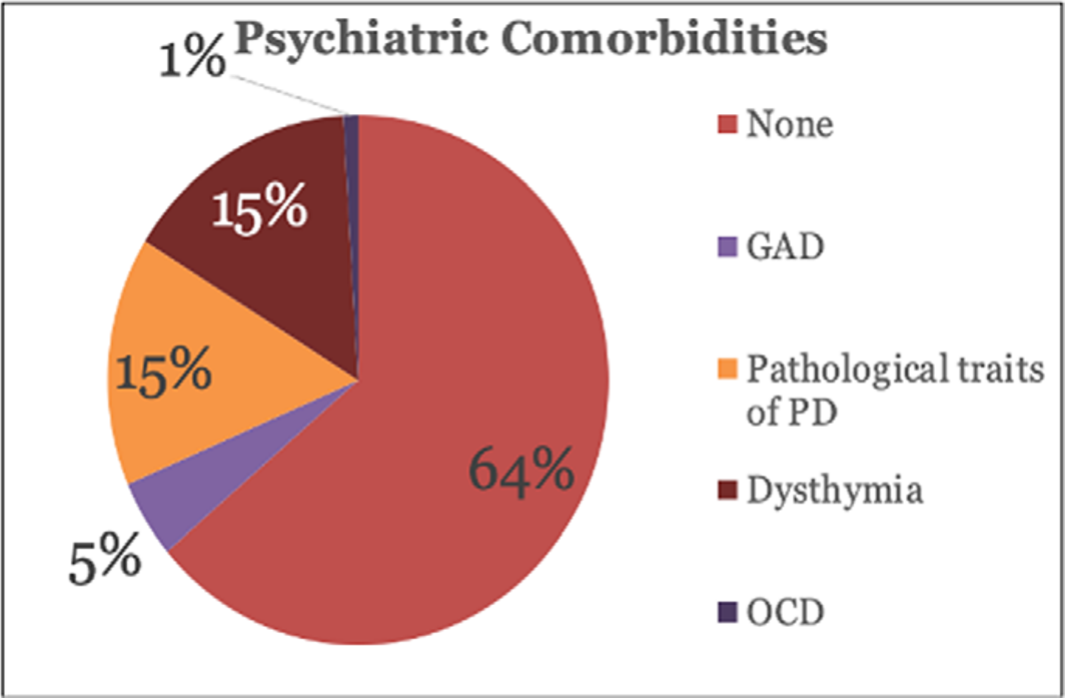

**Image 2:**

**Figure:**
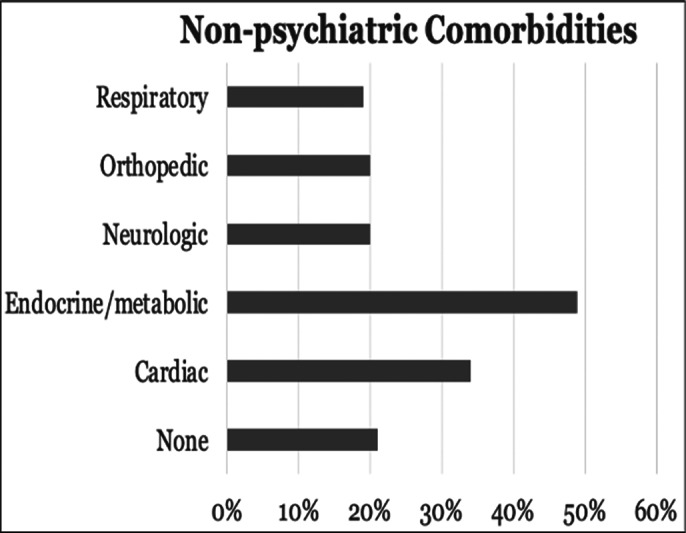

**Image 3:**

**Figure:**
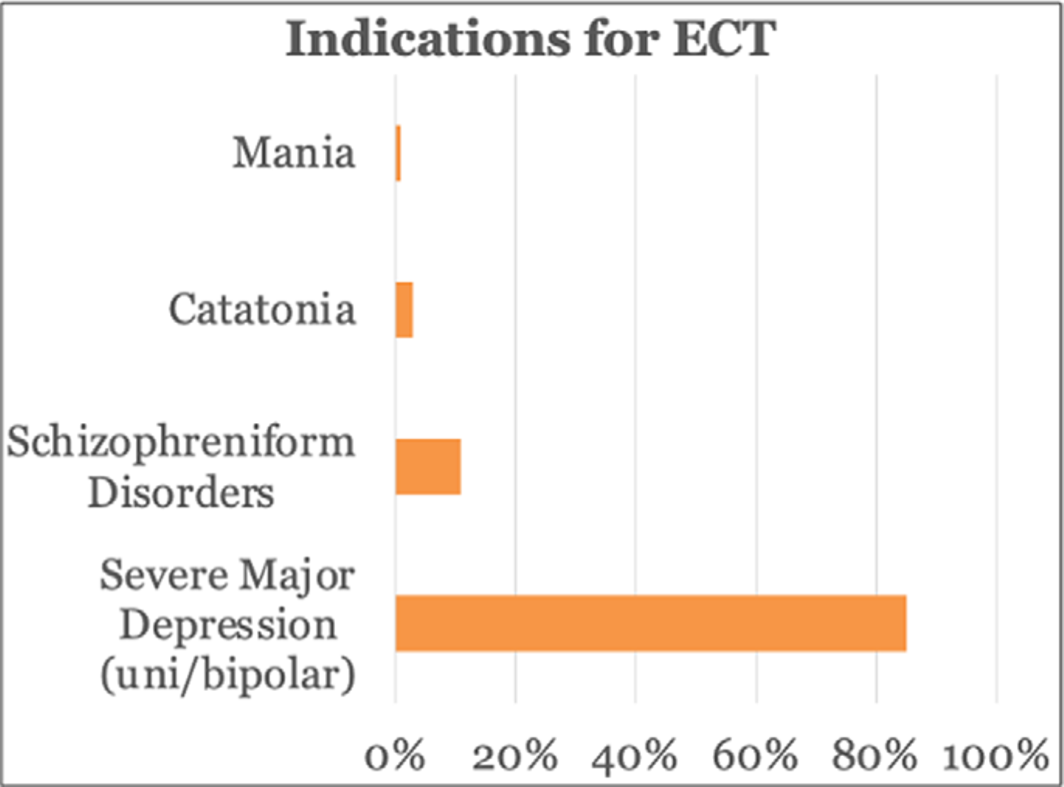

**Conclusions:**

Our sample revealed that the **primary indications for ECT were affective and psychotic disorders**, with a predominant impact on **adult women**. This supports its role as a key intervention for **treatment-resistant conditions**, a finding consistent with existing literature (Leiknes et al., 2012). These **preliminary results** represent an initial evaluation in a broader study aimed at exploring additional aspects of clinical response and comparing ECT with other treatment modalities.

**Disclosure of Interest:**

None Declared

